# Implementation behavior of communities regarding relatives caring for people with dementia: A quantitative study among German communities

**DOI:** 10.1007/s00391-023-02232-w

**Published:** 2023-09-06

**Authors:** Maren Wittek, Fabian Manke-Reimers, Eric Schmitt

**Affiliations:** 1 Institute of Gerontology, Ruprecht-Karls-University of Heidelberg, Bergheimer Straße 20, 69115 Heidelberg, Germany; 2 Center for Preventive Medicine and Digital Health (CPD), Medical Faculty Mannheim, Ruprecht-Karls-University of Heidelberg, Alte Brauerei, Röngtenstraße 7, 68167 Mannheim, Germany

**Keywords:** Cross-sectional study, Questionnaire, Aged, Quantitative study, Support services, Querschnittsstudie, Fragebogen, Ältere Menschen, Quantitative Studie, Unterstützungsmaßnahmen

## Abstract

**Background:**

Actors from the community (CAs) play a fundamental role in the support of caring relatives (CRs) of people with dementia (PWD). As their support is not sufficient, the implementation of support services needs to be optimized; however, little is known about the factors associated with the implementation behavior of CAs.

**Aim:**

This study aimed to investigate the association of person-related factors with the implementation behavior of CAs.

**Methods:**

In a cross-sectional study, 205 CAs from 16 German communities were surveyed with the community implementation behaviour questionnaire (CIBQ), which is based on the theoretical domains framework. Logistic regression analyses were conducted to identify person-related factors associated with the implementation behavior regarding support services for CRs of PWD.

**Results:**

Implementing support services for CRs of PWD is positively and significantly (*p* < 0.001) associated with the agreement of CAs with the CIBQ domains. Higher scores in the CIBQ increase the chance that CAs implemented support services for the target group.

**Conclusion:**

The CIBQ is a tool to determine the status of implementation behavior of communities. This enables an analysis of the areas CAs need to work on in order to optimize the implementation of support services for CRs of PWD or other health and care-related topics.

**Supplementary Information:**

The online version of this article (10.1007/s00391-023-02232-w) contains supplementary material, which is available to authorized users.

## Abstract (de)

**Hintergrund:**

Kommunale AkteurInnen (kA) spielen eine grundlegende Rolle in der Unterstützung pflegender Angehöriger (pA) von Menschen mit Demenz (MmD). Da deren Unterstützung nicht ausreichend ist, sollte die Implementierung von Unterstützungsmaßnahmen optimiert werden. Allerdings ist über Faktoren, die mit dem Implementierungsverhalten der kA zusammenhängen, wenig bekannt.

**Ziel:**

Ziel war die Untersuchung des Zusammenhangs von personenbezogenen Faktoren mit dem Implementierungsverhalten von kA.

**Methoden:**

In einer Querschnittsstudie wurden 205 kA aus 16 deutschen Kommunen befragt. Dazu wurde der Community Implementation Behaviour Questionnaire (CIBQ) verwendet, der auf dem Theoretical Domains Framework basiert. Logistische Regressionsanalysen wurden genutzt, um personenbezogene Faktoren zu identifizieren, die mit dem Implementierungsverhalten bei Unterstützungsmaßnahmen für pA von MmD in Verbindung stehen.

**Ergebnisse:**

Die Umsetzung von Unterstützungsmaßnahmen für pA von MmD ist positiv und signifikant (*p*  < 0,001) mit der Übereinstimmung der kA mit den CIBQ-Domänen assoziiert.

Punktzahlen im CIBQ erhöhen die Chance, dass kA Unterstützungsmaßnahmen für die Zielgruppe implementierten.

**Schlussfolgerung:**

Der CIBQ ist ein Instrument zur Bestimmung des Status des Implementierungsverhaltens von Kommunen. Dies ermöglicht eine Analyse der Bereiche, an denen kA arbeiten sollten, um die Implementierung von Unterstützungsmaßnahmen für pA von MmD oder andere gesundheits- und pflegebezogene Themen zu optimieren.

## Introduction

Taking care of people with dementia (PWD) can be burdensome [11]. As there are many PWD who are cared for by relatives at home [10], supporting those caring relatives (CR) is of high relevance. The communities are one of the entities responsible for offering adequate support for CRs [9, 23].

The theoretical domains framework (TDF) was developed to design and control implementation processes in health care [15]. This study used an adapted version of the TDF, the community implementation behavior questionnaire (CIBQ) [25], to examine person-related factors that influence the implementation behavior of actors from the communities (CAs) regarding the support of CRs of PWD.

## Background

Approximately 4.1 million Germans are classified as in need of care according to the German Code of Social Law XI (SGB XI) [10]. The majority (3.3 million) of care receivers are cared for by relatives at home [10]. A particularly burdened group are CRs of PWD, as they face special challenges due to the symptoms of dementia [11]. This group should be of special interest, as 1.7 million people in Germany are currently living with dementia, which is roughly 2% of the population [8].

The chapter on ”Help for the Aged“ (Altenhilfe) (§71 SGB XII) contains possible elder care measures and circumstances under which these should be provided [13, 20]. Assisting older people is a compulsory task of the municipal communities in the area of essential public services but due to e.g., financial resources and priority setting of the communities, the way and extent of support varies greatly [5]. For example, some communities focus on childcare or on economic topics because of their existing knowledge and experiences. As § 71 SGB XII leaves a lot of room for action and interpretation, the extent and content of elder care varies greatly.

To ensure the care of CRs of PWD as comprehensively as possible, CAs such as senior citizen consultants, nurses and volunteers play a central role. The CAs support, advise or take care of CRs and their PWD. In 2017, the World Health Organization (WHO) assigned the communities to support carers [23]. Additionally, in Germany, the communities were explicitly called upon to develop and offer opportunities to support CRs [9] through various measures (e.g., flyers, care offers, conversation groups) [2, 18]. Therefore, it is of interest to investigate different areas influencing the support of CR of PWD, such as CAs, as they can often influence the focus on CRs of PWD within their work. For instance, some sports clubs offer special courses for people with disabilities or other special issues. It might be possible to implement courses for CRs and PWDs as well. Person-related factors of CAs might be associated with their behavior in implementing support services to optimize the implementation. Although the responsibility of the CAs differs according to their role, position, duties etc., they can become active within their possibilities. For example, a mayor is in a position to offer different measures than a volunteer; however, to the best of our knowledge, there is no evidence on the implementation and the implementation behavior of CAs regarding the support of CRs of PWD.

Michie et al. [15] generated the TDF, a validated framework for implementation science, which was adapted to the gerontological context and to the context of CR of PWD, revalidated and named the CIBQ by Wittek et al. [25]. Both frameworks consist of domains according to person-related factors, such as knowledge and emotions, which are associated with the behavior of actors during the implementation of evidence-based interventions, guidelines in health care [14] or dementia guidelines [16] (TDF) and the implementation of support services for CRs of PWD in a community (CIBQ) [25]. The domains of the newly developed CIBQ can be seen in the supplementary file 1 or in a corresponding paper about the psychometric properties of the CIBQ [25].

This study investigates whether the agreement with the CIBQ domains of CAs is associated with their implementation of support services for CRs of PWD.

## Methods

A cross-sectional questionnaire study was conducted. The survey was anonymous and voluntary. It was conducted online using *LimeSurvey*^1^ and participants received no incentives. Data collection was done from October to December 2021. The study considered the Declaration of Helsinki and was approved by the Ethics Committee of Heidelberg University, Faculty of Behavioral and Cultural Studies (2021 1/1-A1).

### Participants

Participants were actors from 16 different German communities. The recruiting took place in the context of the “Town Hall Project”, which aimed to examine the situation of CRs of PWD as well as their support and consideration in communities by conducting so-called town hall meetings [24]. According to defined criteria, such as the region, number of inhabitants, state and reachability, 45 German communities were invited. Of these, 16 communities and their CAs participated in the “Town Hall Project” and were asked to participate in the CIBQ survey as well. The inclusion criteria were that participating CAs were either already working with CRs and/or PWD or could potentially be working with them, were of legal age (≥ 18 years of age) and understood German. To get in touch with those CAs, multipliers from each community were contacted who knew relevant stakeholders in supporting carers of PWD. Appropriate CAs received an e‑mail including a link to the online survey and were assured about the confidential and anonymous handling of their data. After a nonresponse, the CAs were reminded by e‑mail on two occasions at most.

The distribution of the 16 communities as well as the recruitment were described elsewhere [24–26].

### Measurement

The CIBQ items and domains were adapted and translated in accordance to other existing quantitative questionnaires also based on the TDF [4, 6, 14, 15]. Especially, the determinants of implementation behavior questionnaire by Huijg et al. (2014) was considered [14].

After adapting some items (e.g., for better comprehensibility) during the pre-tests and excluding one domain (D7) for further analysis because of poor fitting indicated by Cronbach’s alpha and inter-item correlation, the final CIBQ consisted of 10 domains and 31 items. A definition of each domain as well as the development and piloting of the survey and its psychometric properties can be found in supplementary file 1 and in Wittek et al. (2022) [25]. The survey is based on self-completion and self-reporting. Participants indicated their level of agreement with each of the 31 items on a 7-point Likert scale (1 = strongly disagree; 7 = strongly agree). Besides the information about the domains, the following data were collected (Supplement 2): sex, age, education, state, population of the community (number of inhabitants), profession (formal e.g. physician, nurse; informal e.g. church, sports club), extent of employment (full-time, part-time, voluntary), work experience (years), proportion of content (CRs of PWD) related tasks within the last 2 years (%), implementation of support services for CRs of PWD within the last 2 years (yes/no), importance of content (CRs of PWD) for the field of work and personal importance (ratio scale 1 = no importance at all; 7 = very great importance).

### Data analysis

The data were analyzed using the open-source software R [19]. All the tests were conducted using 95% confidence intervals (CI) with a *p*-value of 0.05. Mean scores and standard deviations were calculated for the domains. Higher means indicate greater agreement with the items. Pearson correlation coefficients examined associations between the domains and the covariates^2^ [7]. Binary logistic regression analysis (BLRA) was used to investigate the associations between the domains (independent variables) and the self-reported implementation of support services within the last 2 years (dependant variable, yes/no). Furthermore, the covariates were included stepwise (forward and backward) into the analysis (Supplement 3). The Akaike information criterion (AIC) was considered to become lower than in the model including all of the relevant variables as covariates [1]. The BLRA was performed by including a weighted sum score in the form of a principal component to identify if and which simplified structure could represent the 10 domains [4]. Furthermore, a BLRA was conducted with each of the individual domains as an independent variable. To describe the model’s fit, Nagelkerke’s R^2^ (N^2^) was calculated [17].

## Results

### Sample characteristics

From 205 invited CAs, 182 participated in the CIBQ (response rate 88.78%). Of the CAs, 70.8% (*n* = 119) were female, and the mean age was 54.40 years (SD ± 11.09 years). The study participants had different levels of education: training, university degree (of applied sciences), PhD. The CAs worked in various occupational fields. Nearly one quarter (24.4%; *n* = 40) worked in counselling for senior citizens. The majority (82.3%; *n* = 139) worked full-time. More than half (55.6%; *n* = 94) had been working with CRs and/or PWD for more than 10 years but less than 10% (6.1%; *n* = 10) were primarily concerned with this topic. The participants rated the importance of the topic of CRs of PWD as 4.22 (SD ± 1.90) on the 7-point ratio scale for the respective occupational fields of the CAs and as 4.83 (SD ± 1.77) for the CAs personally.

Most of the actors worked in Baden-Wuerttemberg (41.4%; *n* = 70). The majority (47.0%; *n* = 79) of CAs worked in medium-sized cities (20,000–50,000 inhabitants).

### Domain analysis

The domain means ranged from 3.45 (D2 skills, SD ± 1.65) to 5.46 (D1 knowledge, SD ± 1.19) and indicated that the CAs are generally in favor of implementing support services for CRs of PWD. The mean of the sum score of all domains (4.33, SD ± 1.25) is also above 3.50 and therefore indicates a positive attitude towards the implementation. In supplement 4, descriptive values for all domains can be seen.

The domains were highly and significantly (α ≤ 0.01) correlated with each other (Supplement 5). The principle component analysis of the CIBQ domains resulted in one component having an eigenvalue of 6.24 indicating that 62.4% of variability in the domains scores can be described with this single factor.^3^ Following Beenstock et al. (2012) [4], we call this principle component propensity to act.

The variable propensity to act has a positive, significant and

- large correlation with importance for the field of work (0.535)
- medium correlation with profession (0.420), personal importance (0.449) and support services (0.475)
- small correlation with proportion of content-related tasks (0.238).

The variable support services has a positive, significant and

- medium correlation with profession (0.326)
- small correlation with age (0.169), proportion of content-related tasks (0.183), importance for the field of work (0.233) and personal importance (0.212).

### Regression analysis

The AIC of the model including the covariates was 160.72. After conducting a stepwise logistic regression, the AIC was 151.05 and only the variables age, education and profession were included as covariates.

The BLRA in Table 1 shows that the chance that CAs implemented support services for CRs of PWD significantly increased (*p* < 0.001) by an odds ratio (OR) of 2.03 (95% CI 1.50–2.85) with a higher propensity to act. The present model yields an N^2^ = 0.378. According to Backhaus et al. (2006) this applies to an acceptable amount of explained variance [3].

**Table 1:** Binary logistic regression analysis of propensity to act and support services

	Support services		
Independent variables	OR	95% CI	*P* value
*Propensity to act*	2.03	1.50–2.85	< 0.001
**Covariates**			
*Profession*			
Formal	2.22	0.95–5.24	0.066
Informal	1	–	
*Age*	1.04	1.00–1.09	0.033
*Education—University degree*			
Yes	2.92	0.91–9.97	0.076
No	1	–	
N^2^	0.378		

*OR* odds ratio, *CI* confidence interval, *N*^*2*^ Nagelkerke’s R^2^

Furthermore, each individual domain was included into a BLRA as the independent variable (Supplement 6) while support services was still the dependent variable and the covariates were the same as in Table 1. Higher scores in 9 out of 10 domains significantly increased the chance that CAs implemented support services for CRs of PWD. For example, the chance that CAs implemented support services for CRs of PWD significantly increased (*p* < 0.001) by an OR of 2.11 (95% CI: 1.51–3.09) the more likely CAs’ were trained for implementing support services.

## Discussion

According to the chapter in the SGB “Altenhilfe” (§ 71 SGB XII), the WHO and the German national dementia strategy (*Nationale Demenzstrategie*), communities are supposed to support carers [18, 23]. Besides structural determinants, such as infrastructure, [27] the Individuals who (could) work with the CRs of PWD play a key role in the implementation of support services. While the present article measures the propensity to act of CAs, Gansefort et al. [12] conducted a similar project. The community readiness model is based on the concept of community capacity building, which summarizes the development and strength of community structures [21]. Instead of asking actors about their personal propensity to act (self-disclosure; quantitative), they conducted interviews about actors’ estimation of the community’s readiness as a whole (third-party disclosure; qualitative) [12, 25]. As the perspectives and methods differ, the approaches can coexist and even complement each other.

The results of the present study demonstrate that CAs’ implementation of support services for CRs of PWD is positively and significantly associated with their agreement with the CIBQ domains or rather their propensity to act. Furthermore, 9 out of the 10 domains are also on their own significantly associated with the implementation behavior of the CAs within the last 2 years, only the domain D1 knowledge is not. As most of the CAs are already working with CRs and/or PWD, the sample might lack heterogeneity, which is indicated by a low standard deviation (see SD of D1 in Supplement 4). Hence, the group might be too similar within D1 for the sample size to yield significant results. Nevertheless, the results show the relevance of CAs in this context. It turns out that the CIBQ is an appropriate tool to evaluate the current implementation practices in communities. Although it was developed for the topic CRs of PWD, it could be used for further gerontological and communal topics after minor adaptions. For example, the implementation behavior of CAs in supporting people with Parkinsonʼs disease or on the topic of accessibility in communities could be examined.

### Implications

As CAs play a central role in supporting CRs of PWD in communities, it is important to know what is associated with their behavior in implementing support services. For instance, the relatively low mean score in D2-skills suggests that the CAs need further training. The relatively high mean score of D5-beliefs about consequences suggests that the CAs know about the (dis)advantages the implementation of support services for CRs of PWD entails.

The CIBQ can, for instance, be used by community networks to determine their status quo and to decide what they could work on in the future to optimize their implementation behavior and the well-being of carers. Even if the optimization of implementation behavior by itself does not solve all problems, addressing and changing person-related factors might be at least a beginning of improvement.

### Strengths and limitations

In health sciences, implementation research commonly focuses on one innovation, intervention or guideline which is evidence based and realized by a specific profession for a specific target population within a specific setting [22]. This is not the case for the present topic. There are plenty of innovations and measures (to be) implemented within the “gerontological social care setting”. Most of them are “experience based” and the setting as well as the involved professions vary widely; however, although the scope of action of the referenced CAs differs, all of them have the possibility to provide support according to their profession and position. In order to serve the needs of this diverse target group and circumstances, a variety of institutions and professions are required. Furthermore, the requirements in communities differ.

This might be the first study investigating the association of CAs’ person-related factors and the implementation of support services for CRs of PWD in communities. In addition, it might be the first adaptation of the TDF [15] to the described context. Recruiting participants within a cooperation project limited the number of CAs and therefore the variance of single domains. In addition, participants were not randomly selected but identified through multipliers from the different communities. This can cause selection bias.

## Conclusion

The analysis showed that CAs’ implementation of support services for CRs of PWD is positively associated with their agreement with the CIBQ domains. Using the CIBQ provides an opportunity to optimize the health and care of CRs of/and PWD in communities on a time-saving manner by identifying determinants that influence CAs’ existing implementation behavior.

## Practical conclusion

- CRs of PWD are often burdened and CAs are (jointly) responsible for supporting them.
- The CIBQ was used to examine CAs’ implementation behavior regarding the support of CRs of PWD.
- The more CAs agreed with the domains, the greater was their support of CRs of PWD.

### Supplementary Information

Supplement 1: Community Implementation Behaviour Questionnaire

Supplement 2: Characteristics of actors from the community

Supplement 3

Supplement 4: Descriptive values of all domains

Supplement 5: Correlations of all study variables

Supplement 6: Binary logistic regressions of assessing association of independent variables (domains) with support services

## Footnotes

1 Version 3.22.1 + 200129, LimeSurvey, Hamburg, Germany.

2 Grouped with < 0.10 as small, < 0.30 as medium and > 0.50 as large correlations.

3 There were no further principle components having an eigenvalue > 1.

## Declarations

For this article no studies with human participants or animals were performed by any of the authors. All studies mentioned were in accordance with the ethical standards indicated in each case.
