# Supplementary material for: Implementation behavior of communities regarding relatives caring for people with dementia: A quantitative study among German communities
Source: Z Gerontol Geriatr. 2023 Sep 6;57(4):296–301. doi: 10.1007/s00391-023-02232-w (PMC11208208; doi:10.1007/s00391-023-02232-w)
Supplement: Supplementary file 1 — Supplement 1: Community Implementation Behaviour Questionnaire [file 391_2023_2232_MOESM1_ESM.docx]

Supplement 1: Community Implementation Behaviour Questionnaire ^a^

|  | **Domain** | **Items** |
| --- | --- | --- |
| **D1** | **Knowledge** | a. I know why it is important to implement support services for caring relatives of people with dementia in the community.  b. I know the goals for the implementation of support services for caring relatives of people with dementia in the community.  c. I know what is expected of me in the implementation of support services for caring relatives of people with dementia in the community. |
| **D2** | **Skills** | a. I have been trained (e.g. in the context of training/ further training/ induction etc.) on the implementation of support services for caring  relatives of people with dementia in the community.  b. I have the skills or the necessary knowledge to implement support services of people with dementia in the community.  c. I am experienced in implementing support services for caring relatives of people with dementia in the community. |
| **D3** | **Social/Professional Role and Identity** | a. The implementation of support services for caring relatives of people with dementia in the community is part of my job description and  my area of responsibility.  b. I see it as part of my remit to implement support services for caring relatives of people with dementia in the community.  c. My role in the implementation of support services for caring relatives of people with dementia in the community is clearly defined for me. |
| **D4** | **Beliefs about Capabilities** | a. I am convinced that I can implement support services for caring relatives of people with dementia in the community.  b. I am convinced that I can implement support services for caring relatives of people with dementia in the community, even if obstacles  arise.  c. I am convinced that I can implement support services for caring relatives of people with dementia in the community, even if caring  relatives of people with dementia are not motivated. |
| **D5** | **Beliefs about Consequences** | a. If I implement support services for caring relatives of people with dementia in the community, the cooperation in my work environment  is strengthened.  b. The implementation of support services for caring relatives of people with dementia in the community is a satisfying task for me.  c. When I implement support services for caring relatives of people with dementia in the community, it helps caring relatives of people  with dementia to be better cared for. |
| **D6** | **Goals** | a. Within the scope of my field of activity, one of my goals is the implementation of support services for caring relatives of people with  dementia in the community.  b. I set myself realistic short-term goals regarding the implementation of support services for caring relatives of people with dementia in  the community.  c. I set myself realistic long-term goals regarding the implementation of support services for caring relatives of people with dementia in  the community. |

Domain definitions were based on definitions from Huijg et al. (2014)(32).

^a^ The questionnaire was developed in German. For the purpose of this article, all items were translated into English.

Additional file 2: Continued

|  | **Domain** | **Items** |
| --- | --- | --- |
| **D7^b^** | **Sociopolitical Context** | a. With the support of the federal government, the states and municipal communities it is possible to implement support services for  caring relatives of people with dementia in the community.  b. With the support of the care insurance it is possible to implement support services for caring relatives of people with dementia in the  community.  c. With the (given) resources (e.g. staffing/funding) it is possible to implement support services for caring relatives of people with dementia  in the community. |
| **D8** | **Social Influences** | a. Most of the people I matter about are in favour of me implementing support services for caring relatives of people with dementia in the  community.  b. People from my work environment (also) implement support services for caring relatives of people with dementia in the community.  c. People from my work environment are helpful in implementing support services for caring relatives of people with dementia in the community. |
| **D9** | **Emotions** | a. When I implement support services for caring relatives of people with dementia in the community, I am optimistic.  b. When I implement support services for caring relatives of people with dementia in the community, I feel comfortable.  c. When I implement support services for caring relatives of people with dementia in the community, I am not insecure.  d. When I implement support services for caring relatives of people with dementia in the community, I am not frustrated. |
| **D10** | **Reinforcement** | a. When I implement support services for caring relatives of people with dementia in the community, it is valued by carers of people with  dementia.  b. When I implement support services for caring relatives of people with dementia in the community, I receive recognition from my work  environment.  c. When I implement support services for caring relatives of people with dementia in the community, I receive recognition from my private  environment. |
| **D11** | **Nature of the Behaviour** | a. The implementation of support services for caring relatives of people with dementia in the community is something that comes naturally  to me (personally).  b. The implementation of support services for caring relatives of people with dementia in the community is something that I pursue on my  own initiative (also independent of my job description/area of responsibility).  c. The implementation of support services for caring relatives of people with dementia in the community is important for me. |

Domain definitions were based on definitions from Huijg et al. (2014)(32).

^b^ D7 needs to be excluded for further analysis because of poor fitting indicated by Cronbach’s alpha and inter-item correlation.
