# Supplementary material for: Implementation behavior of communities regarding relatives caring for people with dementia: A quantitative study among German communities
Source: Z Gerontol Geriatr. 2023 Sep 6;57(4):296–301. doi: 10.1007/s00391-023-02232-w (PMC11208208; doi:10.1007/s00391-023-02232-w)
Supplement: Supplementary file 2 — Supplement 2: Characteristics of actors from the community [file 391_2023_2232_MOESM2_ESM.docx]

Supplement 2: Characteristics of actors from the community

| **Characteristics**^a^ **(n=182)** | **Total**^b^ |
| --- | --- |
|  |  |
| **Sex of actors** |  |
| Female | 70.8 (119) |
| Male | 29.2 (49) |
| **Age of actors** | 54.40±11.09 |
| **Education**^c^ **of actors** |  |
| University degree (yes) | 84.2 (139) |
| University degree (no) | 15.8 (26) |
| **Occupational field of actors** |  |
| Politics | 10.4 (17) |
| Municipal administration (seniors’ work) | 17.1 (28) |
| Consulting | 24.4 (40) |
| Nursing | 4.9 (8) |
| Volunteering | 14.0 (23) |
| Medicine | 6.7 (11) |
| Pharmacy | 1.8 (3) |
| Church | 6.1 (10) |
| Sports | 4.3 (7) |
| Education | 7.9 (13) |
| Culture | 1.8 (3) |
| Living | 0.6 (1) |
| **Extent of employment of actors in the field of caring relatives and/or people with dementia** |  |
| full-time | 82.3 (139) |
| part-time | 0.0 (0) |
| voluntary | 17.8 (30) |
| **Years of work of actors in the field of caring relatives and/or people with dementia** |  |
| 0-2 | 9.5 (16) |
| >2-5 | 16.6 (28) |
| >5-10 | 18.3 (31) |
| >10 | 55.6 (94) |
| **Workload of actors in the field of caring relatives and/or people with dementia** |  |
| >50% | 6.1 (10) |
| <50% | 93.9 (154) |
| **Importance of caring relatives of people with dementia** |  |
| For the field of work | 4.22±1.90 |
| Personal (actors) | 4.83±1.77 |
| **Actors offering support services in the last 2 years** |  |
| Yes | 50.6 (85) |
| No | 49.4 (83) |
| **State** ^d^ |  |
| Baden-Wuerttemberg | 41.4 (70) |
| Bavaria | 5.9 (10) |
| Hesse | 17.2 (29) |
| North Rhine-Westphalia | 6.5 (11) |
| Rhineland-Palatinate | 5.9 (10) |
| Saxony-Anhalt | 5.3 (9) |
| Schleswig-Holstein | 17.8 (30) |
| **Number of inhabitants** ^d^ |  |
| 5.000 < 10.000 | 1.8 (3) |
| 10.000 < 20.000 | 9.5 (16) |
| 20.000 < 50.000 | 47.0 (79) |
| 50.000 < 100.000 | 13.7 (23) |
| 100.000 – 500.000 | 27.4 (46) |
| > 500.000 | 0.6 (1) |
| ^a^Data presented as percentage (number) except for: age of actors; importance of caring relatives and/or people with dementia presented as mean ±standard deviation.  ^b^Data were missing for sex of actors (n=14); age of actors (n=11); education of actors (n=17); occupational field of actors (n=18); extent of employment of actors in the field of caring relatives and/or people with dementia (n=13); years of work of actors in the field of caring relatives and/or people with dementia (n=13); workload of actors in the field of caring relatives and/or people with dementia (n=18); importance of caring relatives and/or people with dementia occupational field (n=15); importance of caring relatives and/or people with dementia personal (actors) (n=19); actors offering support services in the last 2 years (n=14); state (n=13); number of inhabitants (n=14).  ^c^ The education corresponds to the German education system and has been arranged accordingly.  ^d^ No actors from: Berlin, Brandenburg, Bremen, Hamburg, Lower Saxony, Mecklenburg-Western Pomerania, Saarland, Saxony, Thuringia | |
