# Supplementary material for: Implementation behavior of communities regarding relatives caring for people with dementia: A quantitative study among German communities
Source: Z Gerontol Geriatr. 2023 Sep 6;57(4):296–301. doi: 10.1007/s00391-023-02232-w (PMC11208208; doi:10.1007/s00391-023-02232-w)
Supplement: Supplementary file 3 — Supplement 3 [file 391_2023_2232_MOESM3_ESM.docx]

Supplement 3:

For the construction of the regression model, a stepwise (for- and backward) regression analysis was conducted. Stepwise regression is an empirical approach to covariate selection. To apply the for- and backward approach, R-package “MASS” was used^[[1]](#footnote-1)^. The potential covariates were excluded and included step by step to figure out the best prediction model according to the AIC (Akaike Information Criterion)^[[2]](#footnote-2)^. In the following, the used R-code is displayed. As one can see in the beginning, we used data from full records of the variables included in the full model. These are, besides our primary variable principal component (PC), the covariates sex, age, education (edu_stud), profession (prof), proportion of content (CRs of PWD) related tasks within the last two years (work_.), extent of employment (extent), population of the community (ew_new), work experience (work_exp), importance of content (CRs of PWD) for the field of work (import_area), personal importance of content (CRs of PWD) (import_pers).

The full model was used as the basis for the stepwise regression. The command “stepAIC” tests how the AIC changes when a variable is removed or added to the model based on the best possible (the lowest) AIC. It stops when no covariate improves the AIC when removed or added. One interesting nuance in our analysis is that PC seems to be the most important predictor in every step of the analysis which is indicated by the highest AIC when PC would be removed from the model.

# install.packages("MASS")
library(MASS)

# Full model
full.model = glm(ss_sum_new ~ PC + sex + age + edu_stud + prof +
 extent + work_. + work_exp + import_area + import_pers +
 ew_new, data3, family= "binomial")
summary(full.model)

##
## Call:
## glm(formula = ss_sum_new ~ PC + sex + age + edu_stud + prof +
## extent + work_. + work_exp + import_area + import_pers +
## ew_new, family = "binomial", data = data3)
##
## Deviance Residuals:
## Min 1Q Median 3Q Max
## -2.2643 -0.8709 0.3511 0.7877 2.2468
##
## Coefficients:
## Estimate Std. Error z value Pr(>|z|)
## (Intercept) -7.80162 2.10798 -3.701 0.000215 ***
## PC 0.77503 0.20857 3.716 0.000202 ***
## sex -0.27849 0.48067 -0.579 0.562329
## age 0.03599 0.02367 1.521 0.128317
## edu_stud 1.10075 0.67358 1.634 0.102221
## prof 0.91290 0.49310 1.851 0.064120 .
## extent -0.09936 0.64348 -0.154 0.877288
## work_. 1.25256 1.22702 1.021 0.307339
## work_exp 0.03904 0.07759 0.503 0.614854
## import_area -0.18487 0.14782 -1.251 0.211085
## import_pers 0.03298 0.15454 0.213 0.831033
## ew_new 0.44367 0.40058 1.108 0.268041
## ---
## Signif. codes: 0 '***' 0.001 '**' 0.01 '*' 0.05 '.' 0.1 ' ' 1
##
## (Dispersion parameter for binomial family taken to be 1)
##
## Null deviance: 185.90 on 134 degrees of freedom
## Residual deviance: 136.72 on 123 degrees of freedom
## AIC: 160.72
##
## Number of Fisher Scoring iterations: 5

# Stepwise regression model
step.model <- stepAIC(full.model, direction = "both",
 trace = TRUE)

## Start: AIC=160.72
## ss_sum_new ~ PC + sex + age + edu_stud + prof + extent + work_. +
## work_exp + import_area + import_pers + ew_new
##
## Df Deviance AIC
## - extent 1 136.74 158.74
## - import_pers 1 136.76 158.76
## - work_exp 1 136.97 158.97
## - sex 1 137.06 159.06
## - ew_new 1 137.96 159.96
## - work_. 1 137.97 159.97
## - import_area 1 138.34 160.34
## <none> 136.72 160.72
## - age 1 139.09 161.09
## - edu_stud 1 139.53 161.53
## - prof 1 140.20 162.20
## - PC 1 154.75 176.75
##
## Step: AIC=158.74
## ss_sum_new ~ PC + sex + age + edu_stud + prof + work_. + work_exp +
## import_area + import_pers + ew_new
##
## Df Deviance AIC
## - import_pers 1 136.79 156.79
## - work_exp 1 136.99 156.99
## - sex 1 137.08 157.08
## - ew_new 1 137.96 157.96
## - work_. 1 137.99 157.99
## - import_area 1 138.36 158.36
## <none> 136.74 158.74
## - edu_stud 1 139.53 159.53
## - age 1 139.73 159.73
## - prof 1 140.29 160.29
## + extent 1 136.72 160.72
## - PC 1 154.75 174.75
##
## Step: AIC=156.79
## ss_sum_new ~ PC + sex + age + edu_stud + prof + work_. + work_exp +
## import_area + ew_new
##
## Df Deviance AIC
## - work_exp 1 137.05 155.05
## - sex 1 137.09 155.09
## - work_. 1 138.02 156.02
## - ew_new 1 138.07 156.07
## - import_area 1 138.38 156.38
## <none> 136.79 156.79
## - edu_stud 1 139.53 157.53
## - age 1 139.79 157.79
## - prof 1 140.31 158.31
## + import_pers 1 136.74 158.74
## + extent 1 136.76 158.76
## - PC 1 159.41 177.41
##
## Step: AIC=155.05
## ss_sum_new ~ PC + sex + age + edu_stud + prof + work_. + import_area +
## ew_new
##
## Df Deviance AIC
## - sex 1 137.46 153.46
## - ew_new 1 138.16 154.16
## - work_. 1 138.30 154.30
## - import_area 1 138.64 154.64
## <none> 137.05 155.05
## - edu_stud 1 139.60 155.60
## + work_exp 1 136.79 156.79
## + import_pers 1 136.99 156.99
## - prof 1 141.00 157.00
## + extent 1 137.03 157.03
## - age 1 141.58 157.58
## - PC 1 159.61 175.61
##
## Step: AIC=153.46
## ss_sum_new ~ PC + age + edu_stud + prof + work_. + import_area +
## ew_new
##
## Df Deviance AIC
## - ew_new 1 138.57 152.57
## - work_. 1 138.66 152.66
## - import_area 1 139.09 153.09
## <none> 137.46 153.46
## - edu_stud 1 140.31 154.31
## + sex 1 137.05 155.05
## + work_exp 1 137.09 155.09
## - prof 1 141.16 155.16
## + import_pers 1 137.45 155.45
## + extent 1 137.46 155.46
## - age 1 142.63 156.63
## - PC 1 159.80 173.80
##
## Step: AIC=152.57
## ss_sum_new ~ PC + age + edu_stud + prof + work_. + import_area
##
## Df Deviance AIC
## - import_area 1 139.90 151.90
## - work_. 1 140.27 152.27
## <none> 138.57 152.57
## + ew_new 1 137.46 153.46
## + sex 1 138.16 154.16
## - edu_stud 1 142.16 154.16
## - prof 1 142.19 154.19
## + work_exp 1 138.41 154.41
## + import_pers 1 138.53 154.53
## + extent 1 138.57 154.57
## - age 1 143.60 155.60
## - PC 1 160.48 172.48
##
## Step: AIC=151.9
## ss_sum_new ~ PC + age + edu_stud + prof + work_.
##
## Df Deviance AIC
## - work_. 1 141.05 151.05
## <none> 139.90 151.90
## + import_area 1 138.57 152.57
## - prof 1 142.78 152.78
## + ew_new 1 139.09 153.09
## + sex 1 139.47 153.47
## - edu_stud 1 143.69 153.69
## + work_exp 1 139.71 153.71
## + import_pers 1 139.89 153.89
## + extent 1 139.90 153.90
## - age 1 144.43 154.43
## - PC 1 161.67 171.67
##
## Step: AIC=151.05
## ss_sum_new ~ PC + age + edu_stud + prof
##
## Df Deviance AIC
## <none> 141.05 151.05
## + ew_new 1 139.86 151.86
## + work_. 1 139.90 151.90
## + import_area 1 140.27 152.27
## - edu_stud 1 144.31 152.31
## - prof 1 144.42 152.42
## + sex 1 140.70 152.70
## + work_exp 1 140.88 152.88
## + import_pers 1 141.03 153.03
## + extent 1 141.05 153.05
## - age 1 145.77 153.77
## - PC 1 165.15 173.15

summary(step.model)

##
## Call:
## glm(formula = ss_sum_new ~ PC + age + edu_stud + prof, family = "binomial",
## data = data3)
##
## Deviance Residuals:
## Min 1Q Median 3Q Max
## -2.1589 -0.8952 0.3903 0.8700 2.0728
##
## Coefficients:
## Estimate Std. Error z value Pr(>|z|)
## (Intercept) -7.24478 1.63317 -4.436 9.16e-06 ***
## PC 0.70800 0.16213 4.367 1.26e-05 ***
## age 0.04173 0.01957 2.133 0.0329 *
## edu_stud 1.07141 0.60366 1.775 0.0759 .
## prof 0.79733 0.43419 1.836 0.0663 .
## ---
## Signif. codes: 0 '***' 0.001 '**' 0.01 '*' 0.05 '.' 0.1 ' ' 1
##
## (Dispersion parameter for binomial family taken to be 1)
##
## Null deviance: 185.90 on 134 degrees of freedom
## Residual deviance: 141.05 on 130 degrees of freedom
## AIC: 151.05
##
## Number of Fisher Scoring iterations: 4

1. Ripley B, Venables B, Bates DM, Hornik K, Gebhardt A, Firth D (2023) Support Functions and Datasets for Venables and Ripley's MASS. <http://www.stats.ox.ac.uk/pub/MASS4/> (online, 15.07.2023). [↑](#footnote-ref-1)
2. Akaike H (1973) Information theory and an extension of the maximum likelihood principle. In: Petrov BN, Csaki F (eds) Second international symposium on information theory. Akademiai Kiado, Budapest, p 267-281. [↑](#footnote-ref-2)
