# Supplementary material for: Implementation behavior of communities regarding relatives caring for people with dementia: A quantitative study among German communities
Source: Z Gerontol Geriatr. 2023 Sep 6;57(4):296–301. doi: 10.1007/s00391-023-02232-w (PMC11208208; doi:10.1007/s00391-023-02232-w)
Supplement: Supplementary file 4 — Supplement 4: Descriptive values of all domains [file 391_2023_2232_MOESM4_ESM.docx]

Supplement 4: Descriptive values of all domains

|  | D1 | D2 | D3 | D4 | D5 | D6 | D8 | D9 | D10 | D11 | Domains (sum) |
| --- | --- | --- | --- | --- | --- | --- | --- | --- | --- | --- | --- |
| Mean | 5.46 | 3.45 | 3.52 | 3.82 | 4.61 | 4.14 | 4.31 | 4.47 | 4.63 | 4.82 | 4.33 |
| SD | 1.19 | 1.65 | 1.90 | 1.56 | 1.57 | 1.73 | 1.65 | 1.47 | 1.47 | 1.65 | 1.25 |
| SD = standard deviation  Data were missing for: D1 = Knowledge (n=16); D2 = Skills (n=22); D3 = Social/Professional Role and Identity (n=23); D4 = Beliefs about capabilities (n=24); D5 = Beliefs about Consequences (n=27); D6 = Goals (n=29); D8 = Social Influences (n=29); D9 = Emotions (n=29); D10 = Reinforcement (n=30); D11 = Nature of the Behaviour (n=31); Domains sum (n=31). | | | | | | | | | | | |
