# Supplementary material for: Implementation behavior of communities regarding relatives caring for people with dementia: A quantitative study among German communities
Source: Z Gerontol Geriatr. 2023 Sep 6;57(4):296–301. doi: 10.1007/s00391-023-02232-w (PMC11208208; doi:10.1007/s00391-023-02232-w)
Supplement: Supplementary file 5 — Supplement 5: Correlations of all study variables [file 391_2023_2232_MOESM5_ESM.docx]

Supplement 5: Correlations of all study variables

|  | **1** | **2** | **3** | **4** | **5** | **6** | **7** | **8** | **9** | **10** | **11** | **12** | **13** | **14** | **15** | **16** | **17** | **18** | **19** | **20** | **21** | **22** |
| --- | --- | --- | --- | --- | --- | --- | --- | --- | --- | --- | --- | --- | --- | --- | --- | --- | --- | --- | --- | --- | --- | --- |
| **1** Propensity to act |  | .631^***^ | .785^***^ | .848^***^ | .789^***^ | .856^***^ | .876^***^ | .822^***^ | .760^***^ | .687^***^ | .811^***^ | .143 | -.004 | -.070 | .420^***^ | .238^**^ | .119 | .054 | .059 | .535^***^ | .449^***^ | .475^***^ |
| **2** D1 |  |  | .524^***^ | .553^***^ | .469^***^ | .460^***^ | .506^***^ | .465^***^ | .452^***^ | .298^***^ | .451^***^ | .055 | .107 | -.021 | .267^**^ | .103 | -.022 | .094 | -.067 | .376^***^ | .312^***^ | .223^**^ |
| **3** D2 |  |  |  | .709^***^ | .595^***^ | .642^***^ | .650^***^ | .625^***^ | .453^***^ | .434^***^ | .518^***^ | .225^**^ | .067 | -.149 | .533^***^ | .331^***^ | .058 | .206^*^ | .076 | .455^***^ | .355^***^ | .462^***^ |
| **4** D3 |  |  |  |  | .708^***^ | .677^***^ | .746^***^ | .667^***^ | .535^***^ | .409^***^ | .558^***^ | .081 | -.032 | -.004 | .418^***^ | .254^**^ | .204^*^ | .025 | .158 | .522^***^ | .388^***^ | .463^***^ |
| **5** D4 |  |  |  |  |  | .598^***^ | .743^***^ | .504^***^ | .603^***^ | .387^***^ | .566^***^ | .032 | .033 | -.061 | .269^**^ | .099 | -.012 | .046 | -.029 | .336^***^ | .293^***^ | .290^***^ |
| **6** D5 |  |  |  |  |  |  | .738^***^ | .680^***^ | .576^***^ | .637^***^ | .690^***^ | .173^*^ | -.057 | -.065 | .455^***^ | .189^*^ | .157 | .025 | -.008 | .484^***^ | .410^***^ | .477^***^ |
| **7** D6 |  |  |  |  |  |  |  | .647^***^ | .629^***^ | .530^***^ | .635^***^ | .136 | -.054 | -.077 | .378^***^ | .167 | .044 | .028 | .119 | .482^***^ | .420^***^ | .376^***^ |
| **8** D8 |  |  |  |  |  |  |  |  | .571^***^ | .640^***^ | .657^***^ | .075 | -.104 | -.039 | .328^***^ | .222^**^ | .173^*^ | .008 | .045 | .404^***^ | .322^***^ | .417^***^ |
| **9** D9 |  |  |  |  |  |  |  |  |  | .543^***^ | .695^***^ | .123 | -.018 | -.008 | .209^*^ | .176^*^ | .055 | -.077 | .052 | .365^***^ | .316^***^ | .306^***^ |
| **10** D10 |  |  |  |  |  |  |  |  |  |  | .633^***^ | .118 | -.006 | -.041 | .252^**^ | .154 | .119 | -.030 | .009 | .393^***^ | .252^**^ | .318^***^ |
| **11** D11 |  |  |  |  |  |  |  |  |  |  |  | .107 | .098 | -.080 | .163 | .152 | .095 | .104 | .027 | .390^***^ | .449^***^ | .353^***^ |
| **12** Sex |  |  |  |  |  |  |  |  |  |  |  |  | -.206^*^ | -.140 | .156 | .119 | .101 | -.167 | .010 | .108 | .200^*^ | -.014 |
| **13** Age |  |  |  |  |  |  |  |  |  |  |  |  |  | .067 | .008 | .059 | -.413^***^ | .404^***^ | .012 | .052 | .020 | .169 |
| **14** Education |  |  |  |  |  |  |  |  |  |  |  |  |  |  | -.062 | -.148 | .003 | -.120 | .119 | -.096 | -.155 | .103 |
| **15** Profession |  |  |  |  |  |  |  |  |  |  |  |  |  |  |  | .180^*^ | .233^**^ | .145 | .083 | .360^***^ | .089 | .326^***^ |
| **16** Proportion of content-related tasks |  |  |  |  |  |  |  |  |  |  |  |  |  |  |  |  | .033 | .096 | .127 | .334^***^ | .187^*^ | .183^*^ |
| **17** Extent of employment |  |  |  |  |  |  |  |  |  |  |  |  |  |  |  |  |  | -.105 | .056 | .079 | -.017 | .021 |
| **18** Years of work |  |  |  |  |  |  |  |  |  |  |  |  |  |  |  |  |  |  | -.143 | .096 | .061 | .126 |
| **19** Population |  |  |  |  |  |  |  |  |  |  |  |  |  |  |  |  |  |  |  | .121 | .118 | .139 |
| **20** Importance – for the field of work |  |  |  |  |  |  |  |  |  |  |  |  |  |  |  |  |  |  |  |  | .310^***^ | .233^**^ |
| **21** Importance - personal |  |  |  |  |  |  |  |  |  |  |  |  |  |  |  |  |  |  |  |  |  | .212^*^ |
| **22** Support services |  |  |  |  |  |  |  |  |  |  |  |  |  |  |  |  |  |  |  |  |  |  |

*p ≤ .05; **p ≤ .01, ***p < .001.

D1 = Knowledge; D2 = Skills; D3 = Social/Professional Role and Identity; D4 = Beliefs about capabilities; D5 = Beliefs about Consequences; D6 = Goals; D8 = Social Influences; D9 = Emotions; D10 = Reinforcement; D11 = Nature of the Behaviour.
