# Supplementary material for: Implementation behavior of communities regarding relatives caring for people with dementia: A quantitative study among German communities
Source: Z Gerontol Geriatr. 2023 Sep 6;57(4):296–301. doi: 10.1007/s00391-023-02232-w (PMC11208208; doi:10.1007/s00391-023-02232-w)
Supplement: Supplementary file 6 — Supplement 6: Binary logistic regressions of assessing association of independent variables (domains) with support services [file 391_2023_2232_MOESM6_ESM.docx]

Supplement 6: Binary logistic regressions of assessing association of independent variables (domains) with *support services*

|  | **Support Services** | | | |
| --- | --- | --- | --- | --- |
| **Independent variables^a^** | OR | 95% CI | P value | N² |
| Propensity to act | 2.03 | 1.50 – 2.85 | <.001^***^ | .378 |
| D1 – Knowledge | 1.29 | 0.94 – 1.80 | .127 | .211 |
| D2 – Skills | 2.11 | 1.51 – 3.09 | <.001^***^ | .358 |
| D3 – Social/Professional Role and Identity | 1.72 | 1.36 – 2.25 | <.001^***^ | .361 |
| D4 – Beliefs about Capabilities  Beliefs about Consequences  Goals | 1.42 | 1.10 – 1.87 | .009^**^ | .250 |
| D5 – Beliefs about Consequences  Beliefs about Consequences  Goals | 2.18 | 1.57 – 3.19 | <.001^***^ | .387 |
| D6 – Goals | 1.59 | 1.23 – 2.09 | <.001^***^ | .300 |
| D8 – Social Influences | 1.83 | 1.39 – 2.49 | <.001^***^ | .351 |
| D9 – Emotions | 1.52 | 1.16 – 2.03 | .003^**^ | .269 |
| D10 – Reinforcement | 1.58 | 1.19 – 2.16 | .002^**^ | .274 |
| D11 – Nature of the Behaviour | 1.62 | 1.26 – 2.12 | <.001^***^ | .310 |
| OR = Odds Ratio; CI = Confidence Interval; **p ≤ .05; *p ≤ .01.; N² = Nagelkerke’s R²/ adjusted R²  Included covariates: profession; proportion of content-related (caring relatives and/or people with dementia) tasks; occupational tasks before/during implementation; Importance for the field of work/personal. | | | | |
